# Suppressed oncogenic molecules involved in the treatment of colorectal cancer by fecal microbiota transplantation

**DOI:** 10.3389/fmicb.2024.1451303

**Published:** 2024-11-13

**Authors:** Xing Han, Bo-Wen Zhang, Wei Zeng, Meng-Lin Ma, Ke-Xin Wang, Bao-Juan Yuan, Dan-Qi Xu, Jia-Xin Geng, Chao-Yuan Fan, Zhan-Kui Gao, Muhammad Arshad, Shan Gao, Liangliang Zhao, Shu-Lin Liu, Xiao-Qin Mu

**Affiliations:** ^1^Genomics Research Center (Key Laboratory of Gut Microbiota and Pharmacogenomics of Heilongjiang Province), College of Pharmacy, Harbin Medical University, Harbin, China; ^2^National Key Laboratory of Frigid Zone Cardiovascular Diseases, Harbin Medical University, Harbin, China; ^3^HMU-UCCSM Centre for Infection and Genomics, Harbin Medical University, Harbin, China; ^4^Pathology Department, The Second Affiliated Hospital of Harbin Medical University, Harbin, China; ^5^Department of Colorectal Surgery, First Affiliated Hospital of Harbin Medical University, Harbin, China; ^6^Department of Microbiology, Immunology and Infectious Diseases, University of Calgary, Calgary, AB, Canada; ^7^Translational Medicine Research and Cooperation Center of Northern China, Heilongjiang Academy of Medical Sciences, Harbin, China

**Keywords:** colorectal cancer, intestinal microbiota, intestinal homeostasis, fecal microbiota transplantation, oncogenic molecules

## Abstract

Dysbiosis of the intestinal microbiota is prevalent among patients with colorectal cancer (CRC). This study aims to explore the anticancer roles of the fecal microbiota in inhibiting the progression of colorectal cancer and possible mechanisms. The intestinal microbial dysbiosis in CRC mice was significantly ameliorated by fecal microbiota transplantation (FMT), as indicated by the restored ACE index and Shannon index. The diameter and number of cancerous foci were significantly decreased in CRC mice treated with FMT, along with the restoration of the intestinal mucosal structure and the lessening of the gland arrangement disorder. Key factors in oxidative stress (TXN1, TXNRD1, and HIF-1α); cell cycle regulators (IGF-1, BIRC5, CDK8, HDAC2, EGFR, and CTSL); and a critical transcription factor of the innate immune signal pathway (IRF5) were among the repressed oncogenic targets engaged in the FMT treatment of CRC. Correlation analysis revealed that their expressions were positively correlated with uncultured_bacterium_o_Mollicutes_RF39, Rikenellaceae_RC9_gut_group, and negatively correlated with *Bacillus*, *Marvinbryantia*, *Roseburia*, *Angelakisella*, *Enterorhabdus*, *Bacteroides*, *Muribaculum*, and genera of uncultured_bacterium_f_Eggerthellaceae, uncultured_bacterium_f_Xanthobacteraceae, Prevotellaceae_UCG-001, uncultured_bacterium_f_Erysipelotrichaceae, uncul-tured_bacterium_f_Lachnospiraceae, uncultured_bacterium_f_Ruminococcaceae, Eubacterium_coprostanoligenes_group, Ruminococcaceae_UCG-005, and uncultured_bacterium_f_Peptococcaceae. This study provides more evidence for the application of FMT in the clinical treatment of CRC.

## 1 Introduction

The incidence and mortality of colorectal cancer (CRC) are 9.3 and 9.6%, respectively, in 2022, according to data on the global cancer burden provided by the International Agency for Research on Cancer. Conventional cancer treatments include radiation, chemotherapy, and surgery (Ossibi et al., 2022). However, 66% of CRC patients who have undergone radical surgical resection have developed recurrent and metastatic carcinoma (Plazas et al., 2015). Patients’ survival rates can be greatly increased by adjuvant chemotherapy, which includes fluorouracil, oxaliplatin, capecitabine, irinotecan, and panitumumab. On the other hand, the adverse effects—which might include heart toxicity, gastrointestinal upset, and suppression of the bone marrow—can be extremely painful (Chen et al., 2021; Gobran et al., 2020; Huang et al., 2016; Lam and Lee, 2012). Investigating a secure and efficient CRC treatment strategy is therefore essential.

The human gut is home to over a thousand different types of bacteria that coexist in a dynamic balance and are an essential component of the physiological ecosystem (Jiang et al., 2020). Intestinal microbiota is responsible for preserving the homeostasis of the internal milieu, fostering immune system maturation, preventing the incursion of harmful microbes, and providing the host with biochemical metabolic pathways and enzymes absent in humans (Jiang et al., 2020; Monreal et al., 2005). However, it is evident that nutrition, exercise, and stress all have an impact on the diversity and abundance of the intestinal microbiota (Gubert et al., 2020). Once the balance of the gut microbiota is disturbed, inflammation is induced and carcinogenic pathways are activated, which leads to a disordered intestinal micro-ecology and the development of colorectal cancer (Tilg et al., 2018). Therefore, maintaining the balance of the intestinal microbiota might be an effective strategy for slowing the progression of CRC.

Fecal microbiota transplantation (FMT) offers significant potential for treating CRC by reversing intestinal microbial dysbiosis (Kaźmierczak-Siedlecka et al., 2020). FMT provides *Lactobacillus rhamnosus* and *Lactobacillus plantarum*, which stimulate mucin production and enhance intestinal barrier function (Fong et al., 2020). Short-chain fatty acids (SCFAs) are important microbial metabolites in the gut and are mainly composed of acetate, propionate, and butyrate. FMT regulates the production of SCFAs by inhibiting NF-κB reporter activity and thus alleviating CRC (Zhang et al., 2020). FMT increases the abundance of *Bacteroidetes thetaiotaomicron*, which subsequently induces immune responses in dendritic cells and mediates gut homeostasis (Huang et al., 2022).

Many studies have demonstrated the importance of FMT in restoring the balance of the gut microbiota, but little is known on its anticancer properties. In this study, we screened pathways that were reported to be associated with carcinogenesis, identified 10 potential targets for FMT and predicted the gut bacteria associated with each target. This study provides support to the application of FMT in CRC treatment.

## 2 Materials and methods

### 2.1 The CRC mouse model

Male Balb/c mice (8-week-old) were purchased from Beijing Vital River Lab. All animals had free access to food and water while living in ventilated cages with 12-h light/dark cycles. The CRC mouse model was established by using the carcinogen azoxymethane (AOM, Sigma) and the proinflammatory agent dextran sulfate sodium (DSS, MP Biomedicals) as previously described (Yu et al., 2023). To put it briefly, AOM (10 mg/kg) was injected intraperitoneally into the mice during the first week of model establishment. The mice were allowed to drink water containing 2.5% DSS during the second week. The mice were provided unrestricted access to sterile water without DSS throughout the third and fourth weeks. Two more cycles of the treatments from week 2 to week 4 were conducted. Following the establishment of the CRC model, mice were randomized into two experimental groups (AOM/DSS and AOM/DSS + FMT), along with one control group without treatment with AOM/DSS and FMT. Each group of mice had five cages with four mice per cage, all of which were raised at random. The experiments were approved by the Ethics Committee of Harbin Medical University (HMUIRB20180013).

### 2.2 Fecal microbiota transplantation

Fresh faeces from mice in the normal control group were collected, mixed with PBS (200 mg/mL), and centrifuged at 1,500 rpm for 1 min to obtain the microbial supernatant. Each recipient mouse in the FMT-treated group was given 1 mL of fecal slurry by enema twice a week beginning in the second week of model establishment and continuing until the end of the study.

### 2.3 16S rDNA amplicon sequencing

Two tubes of feces were collected in each cage, and a total of 10 tubes were collected for each experimental group. The total genomic DNA was isolated from feces by using TIANamp Stool DNA Kit (Tiangen). 16S rDNA sequencing of the fecal microbiota was conducted by Beijing Biomarker Biotechnologies. The V3 and V4 regions of the 16S rDNA were amplified with the specific primers 338F (5′-ACTCCTACGGGAGGCAGCA-3′) and 806R (5′-GGACTACHVGGGTWTCTAAT-3′) and sequenced on the Illumina HiSeq 2500. Sequencing data for six replicates was obtained in each experimental group and submitted to NCBI (PRJNA 949363). Taxonomy was assigned to the OTUs using the SILVA database (v.123) with the RDP classifier at a 70% confidence threshold. The alpha diversity indicated by the ACE index and Shannon index was calculated using QIIME2 software (Version 2020.8). Beta diversity analysis was performed to investigate the structural variation in microbial communities across samples using non-metric multidimensional scaling (NMDS) and principal coordinate analysis (PCoA) by QIIME-1.8.0, and visualized by R 3.1.1. Statistical significance was determined by one-way analysis of variance. Spearman rank correlation analysis was employed to identify data with correlation coefficient larger than 0.1 and *p*-value smaller than 0.05, which were used to construct the network.

### 2.4 HE staining

After being extracted, colorectal tissue was fixed with polyformaldehyde and embedded in paraffin blocks. Sections of 3 mm thickness were cut for staining with hematoxylin for 2 min and with eosin for 1 min. The slices were finally sealed with neutral resin and then imaged.

### 2.5 Immunohistochemistry

After dewaxing in xylene and hydrating in gradient ethanol, the paraffin slices were treated with 3% H_2_O_2_ for 10 min to block endogenous peroxidase. Antigen retrieval was conducted in a sodium citrate solution at high power for 2 min and then at low power for 10 min in the microwave, respectively. Tissues were then blocked at room temperature for 1 h using 50% goat serum. Slices were incubated at 4°C overnight with primary antibodies for ki67 (1:200, 12202, Cell Signaling Technology), cleaved caspase-3 (1:1000, 9661, Cell Signaling Technology), IGF-1 (1:100, A12305, Abclonal), BIRC5 (1:100, GB11177, Servicebio), CDK8 (1:100, A9654, Abclonal), HDAC2 (1:100, A2084, Abclonal), EGFR (1:100, GB111504, Servicebio), TXN1 (1:100, GB11993, Servicebio), TXNRD1 (1:100, A16631, Abclonal), HIF1-α (1:100, A16873, Abclonal), IRF5 (1:100, A16388, Abclonal), or CTSL (1:100, A12200, Abclonal). After washing with PBS, the tissues were incubated with HRP-labeled secondary antibody (1:200, AS014, Abclonal) at 37°C for 1 h. The staining was visualized with a DAB peroxidase substrate kit (Origene). The slices were finally dehydrated in ethanol and xylene and sealed with resin. The tissues were photographed using Olympus microscope and the yellow-brown area indicates a positive expression.

### 2.6 PCR array

The total RNA of the entire colorectal tissue was extracted by using the rneasy plus mini kit (Qiagen) and then converted to cDNA by using the RT2 first strand kit (Qiagen). RT2 profiler PCR array (PAMM-507ZA, Qiagen) was employed to detect the mouse cancer drug targets, including *Abcc1*, *Akt1*, *Akt2*, *Atf2*, *Aurka*, *Aurkb*, *Aurkc*, *Bc12*, *Birc5*, *Cdc25a*, *Cdk1*, *Cdk2*, *Cdk4*, *Cdk5*, *Cdk7*, *Cdk8*, *Cdk9*, *Ctsb*, *Ctsd*, *Ctsl*, *Ctss*, *Egfr*, *Erbb2*, *Erbb3*, *Erbb4*, *Esr1*, *Esr2*, *Figf*, *Flt1*, *Flt4*, *Grb2*, *Gstp1*, *Hdac1*, *Hdac11*, *Hdac2*, *Hdac3*, *Hdac4*, *Hdac6*, *Hdac7*, *Hdac8*, *Hif1a*, *Hras*, *Hsp90aa1*, *Hsp90b1*, *Igf1*, *Igf1r*, *Igf2*, *Irf5*, *Kdr*, *Kit*, *Kras*, *Mdm2*, *Mdm4*, *Mtor*, *Nfkb1*, *Nras*, *Ntn3*, *Parp1*, *Parp2*, *Parp4*, *Pdgfra*, *Pdgfrb*, *Pgr*, *Piksc2a*, *Pik3c3*, *Pik3ca*, *Plk1*, *Plk2*, *Plk3*, *Plk4*, *Prkca*, *Prkcb*, *Prkcd*, *Prkce*, *Ptgs2*, *Rhoa*, *Rhob*, *Tert*, *Tnks*, *Top2a*, *Top2b*, *Trp53*, *Txn1*, *Txnrd1*.

### 2.7 Western blot

To get the protein-containing supernatant, the entire colorectal tissue was homogenized in RIPA lysis buffer supplemented with protease inhibitor PMSF. Tissue proteins were separated through SDS-PAGE electrophoresis and transferred onto a PVDF membrane, followed by blocking with fast blocking buffer (Biosharp) for 10 min, and then incubated overnight at 4°C with primary antibodies: IGF-1 (1:500, A12305, Abclonal), BIRC5 (1:500, GB11177, Servicebio), CDK8 (1:500, A9654, Abclonal), HDAC2 (1:500, A2084, Abclonal), EGFR (1:500, GB111504, Servicebio), TXN1 (GB11993, Servicebio), TXNRD1 (A16631, Abclonal), HIF1-α (1:500, A16873, Abclonal), IRF5 (1:500, A16388, Abclonal), CTSL (1:500, A12200, Abclonal), GAPDH (1:2000, A19056, Abclonal) or β-Tubulin (1:2000, AC008, Abclonal). After washing with PBS, membranes were incubated at room temperature for 2 h with HRP goat anti-rabbit IgG (H + L) (1:5000, AS014, Abclonal). Images were acquired by using Tanon-5200 and Tanon MP.

### 2.8 Statistical analysis

Statistical analysis was performed by Tukey’s test, and the statistical comparison was performed by GraphPad Prism 8.0. All data were expressed as mean ± SD. A two-tailed value of *p* < 0.05 was taken to indicate statistical significance. All experiments were repeated independently at least three times.

## 3 Results

### 3.1 FMT reduces the burden of CRC

**Figure 1:**
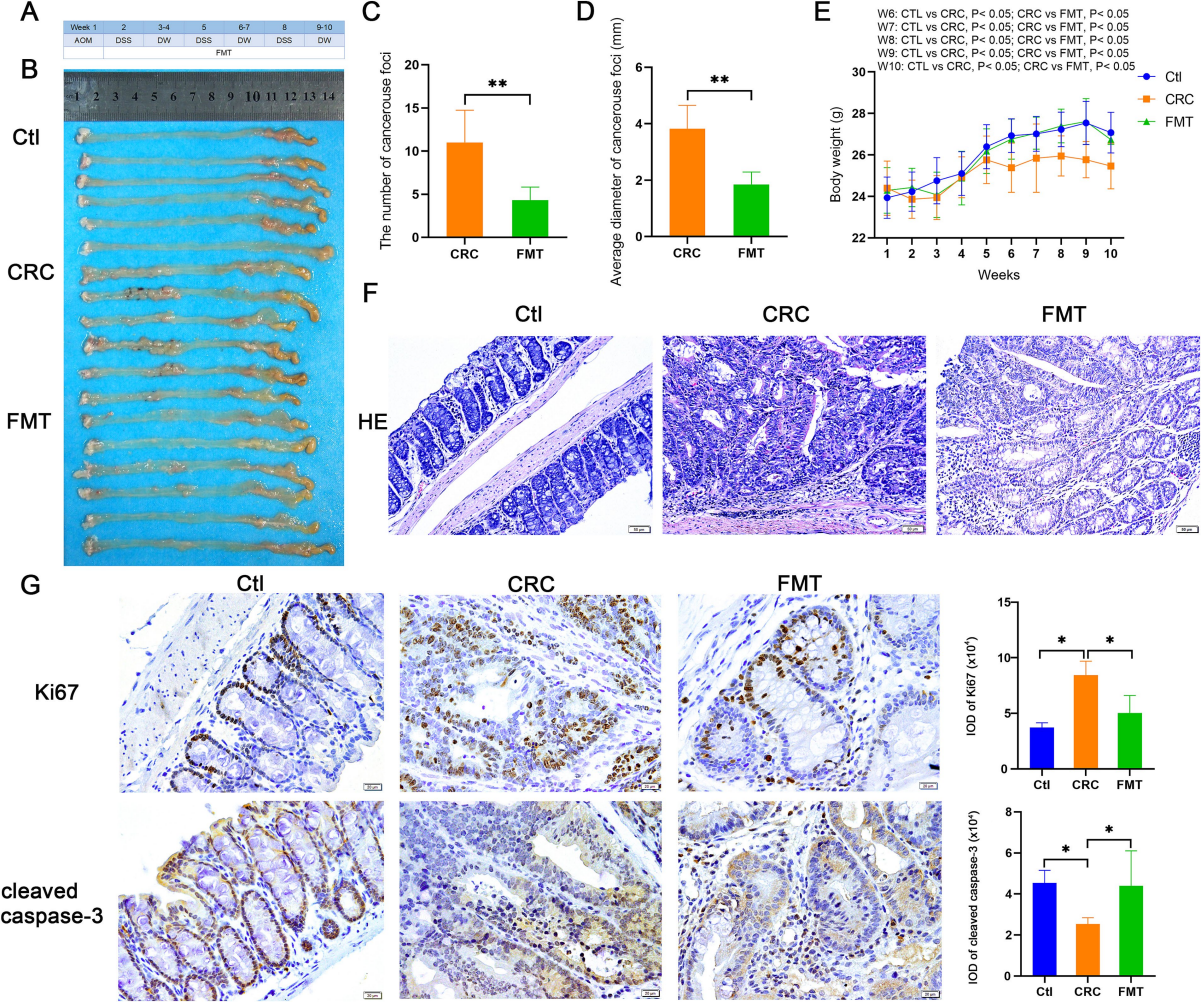
Inhibitory effect of FMT on CRC progression. **(A)** Design of the experiment. **(B)** Pictures of intestinal tissue. Ctl, normal control mice; CRC, colorectal cancer mice; FMT, colorectal cancer mice receiving fecal microbiota transplantation. **(C,D)** The average number and diameter of cancerous foci. ^**^p < 0.01 (Ctl, *n* = 6; CRC, *n* = 6; FMT, *n* = 6). **(E)** The average body weight of mice in each week. ^*^p < 0.05 (Ctl, *n* = 19; CRC, *n* = 15; FMT, *n* = 18). **(F)** HE staining of intestinal tissue. **(G)** Immuno-histochemical staining of Ki67 and cleaved caspase-3 in intestinal tissue. ^*^p < 0.05, *n* = 6.

Following a 10-week period of establishment of the CRC mouse model, a significant number of malignant foci were identified in the intestines of CRC mice, with an average of 11 and an average diameter of 3.8 mm. Additionally, the expression of ki67 was raised in these foci when compared to the normal control animals. The average number of foci in CRC mice treated with FMT dropped to 4, and their average diameter was 1.9 mm. Increased expression of cleaved caspase-3 indicated that cancer cells were more likely to undergo apoptosis, which was substantially responsible for the reduction of cancer foci (Figures 1A–D,G). Morphologically, the mucosal layer’s structure was damaged in CRC mice, along with the goblet and crypt cells’ absence, a significant infiltration of inflammatory cells, and an abnormal glandular tube. In mice treated with FMT, the lesions were greatly ameliorated, with less destruction of crypt and goblet cells and the glands being regularly arranged (Figure 1F). Body weight indirectly reflects intestinal functions. Compared with mice in the normal control group, at the sixth week of inducing the CRC model, CRC mice began to show significant body weight reduction, while the trend of weight loss was significantly inhibited by FMT (Figure 1E).

### 3.2 FMT reverses intestinal microbial dysbiosis in CRC mice

We used 16S rDNA high-throughput sequencing to detect the diversity of gut microbiota in order to determine the differences in gut microbiota between CRC mice and normal control mice, as well as whether fecal microbiota transplantation may correct these differences. The 16S rDNA of the intestinal microbiota was sequenced using Illumina HiSeq 2500. Following quality control and assembly, a total of 1,777,674 clean reads were collected from 18 samples. The typical length ranges from 415 bp to 422 bp, making it possible to encompass the 16S rDNA’s V3 and V4 sections. The clean reads number for each sample and rarefaction curves were shown in (Supplementary Table 1 and Supplementary Figures 1A,B). OTU clustering was done with a 97% similar sequence. A total of 17 phyla, 29 classes, 57 orders, 94 families, 183 genera, and 199 species were identified among the intestinal bacteria.

**Figure 2:**
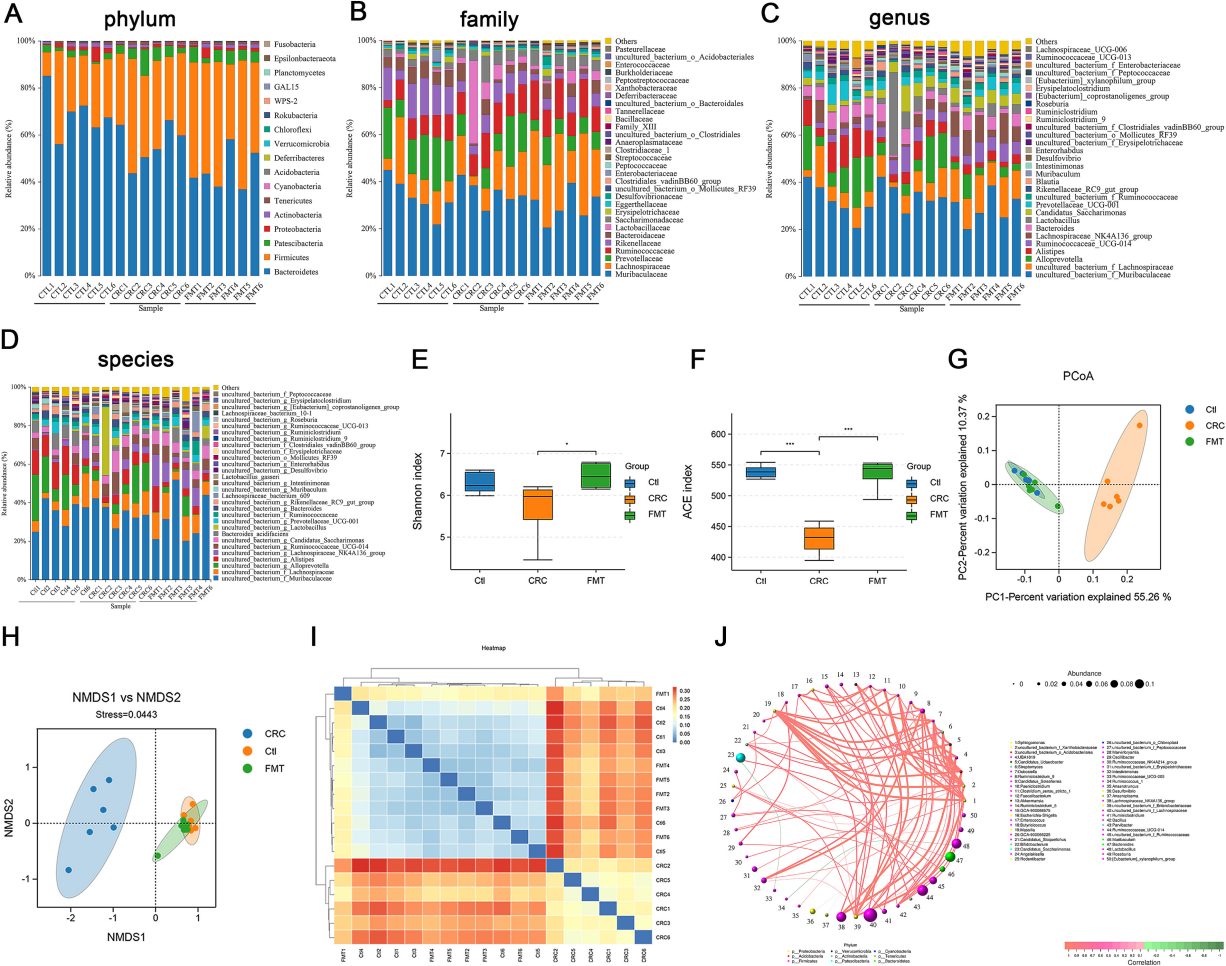
FMT reverses the microbial disturbance in CRC mice. **(A–D)** The distribution of gut microbiota at the levels of phylum, family, genus and species. **(E,F)** Alpha diversity indicated by Shannon index **(E)** and ACE index **(F)**, ^*^p < 0.05 and ^***^p < 0.001, *n* = 6. (G,H) Beta diversity indicated by PCoA analysis **(G)** and NMDS analysis **(H)**. **(I)** Cluster analysis of samples. **(J)** Bacterial co-occurrence network.

The relative abundance of intestinal bacteria in CRC mice was significantly different at the phylum, family, genus and species levels compared to the normal control mice (Figures 2A–D). We first examined the predominant microbes of each group at the genus level. Intestinal bacteria enriched in normal control mice were genera of uncultured_bacterium_f_Muribaculaceae (28.21%), *Alloprevotella* (12.68%), *Alistipes* (9.81%), uncultured_bacterium_f_Lachnospiraceae (8.69%), *Bacteroides* (5.60%), Prevotellaceae_UCG-001 (4.68%), Ruminococcaceae_UCG-014 (4.10%), Lachnospiraceae_NK4A136_group (2.79%), uncultured_bacterium_f_Ruminococcaceae (2.43%), *Muribaculum* (1.09%), and *Enterorhabdus* (1.06%). Some of these microbes, such as *Alloprevotella* (8.31%), *Alistipes* (4.92%), *Bacteroides* (3.81%), Prevotellaceae_UCG-001 (2.18%), uncultured_bacterium_f_Ruminococcaceae (1.97%), *Muribaculum* (0.62%), and *Enterorhabdus* (0.69%) showed a notable decline in percentage in CRC mice. Furthermore, as shown in Figure 2C, the intestinal bacteria Rikenellaceae_RC9_gut_group (1.70%), *Blautia* (1.28%), uncultured_bacterium_o_Mollicutes_RF39 (0.85%), and *Intestinimonas* (0.80%) were significantly increased in CRC mice. The FMT therapy significantly reversed the abundance of *Bacteroides* (4.54%), Prevotellaceae_UCG-001 (3.93%), uncultured_bacterium_f_Ruminococcaceae (2.56%), *Enterorhabdus* (1.03%), and uncultured_bacterium_f_Erysipelotrichaceae (0.83%). Certain intestinal bacteria were completely identified at the species level as follows with the percentage of normal controls mice and CRC mice: the increased species of *Lactobacillus_gasseri* (0.10 to 2.19%), *Lachnospiraceae_bacterium_609* (0.48 to 1.16%), *Clostridiales_bacterium_CIEAF_020* (0.008 and 0.014%), *Phaseolus_acutifolius_tepary_bean* (0.009 and 0.013%), and the decreased species of *Bacteroides_acidifaciens* (5.30 to 2.13%), *Lachnospiraceae_bacterium_10-1* (0.26 to 0.21%), *Mucispirillum_schaedleri_ASF457* (0.08 to 0.04%), *Clostridium_sp* (0.03 to 0.01%), *Burkholderiales_bacterium_YL45* (0.02 to 0.01%), and *Lachnospiraceae_bacterium_COE1* (0.08 to 0.01%). The use of FMT reversed most of the alterations, such as *Lactobacillus_gasseri* (0.31%), *Bacteroides_acidifaciens* (3.34%), *Mucispirillum_schaedleri_ASF457* (0.05%), *Clostridium_sp* (0.04%), *Lachnospiraceae_bacterium_COE1* (0.12%).

In addition to examining the dominant microbiota, we assessed the alpha diversity of gut microbiota, including the Shannon index and ACE index, to confirm the improvement of the entire gut microbiota in CRC mice following FMT. In CRC mice, there was a considerable decrease in the Shannon index and ACE index, which indicate the richness and diversity of the microbial population. However, the declining trend was reversed by FMT (Figures 2E,F). To confirm if FMT could effectively lessen the difference in gut microbiota diversity between groups and reverse the disorder of the gut microbiota, we conducted β diversity analysis using non-metric multidimensional scaling (NMDS) and principal coordinate analysis (PCoA). The analysis of PCoA and NMDS revealed that the microbial structure of normal control mice and FMT-treated mice was similar but considerably different from that of the CRC mice (Figures 2G,H). Furthermore, sample clustering analysis of unweighted unifrac demonstrated that the phylogenetic relationship of the intestinal bacteria between mice in the normal control and FMT groups was more closely linked compared to the CRC mice (Figure 2I). These results indicated that FMT considerably reduced the dysbiosis of the gut microbiota, shaping the gut microbiota of CRC mice to resemble that of normal controls. Additionally, through complex network relationships, intestinal bacteria supported or inhibited one another, working together to preserve the homeostasis of intestinal microbiota (Figure 2J).

### 3.3 The suppressed oncogenic targets in the treatment of CRC by FMT

**Figure 3:**
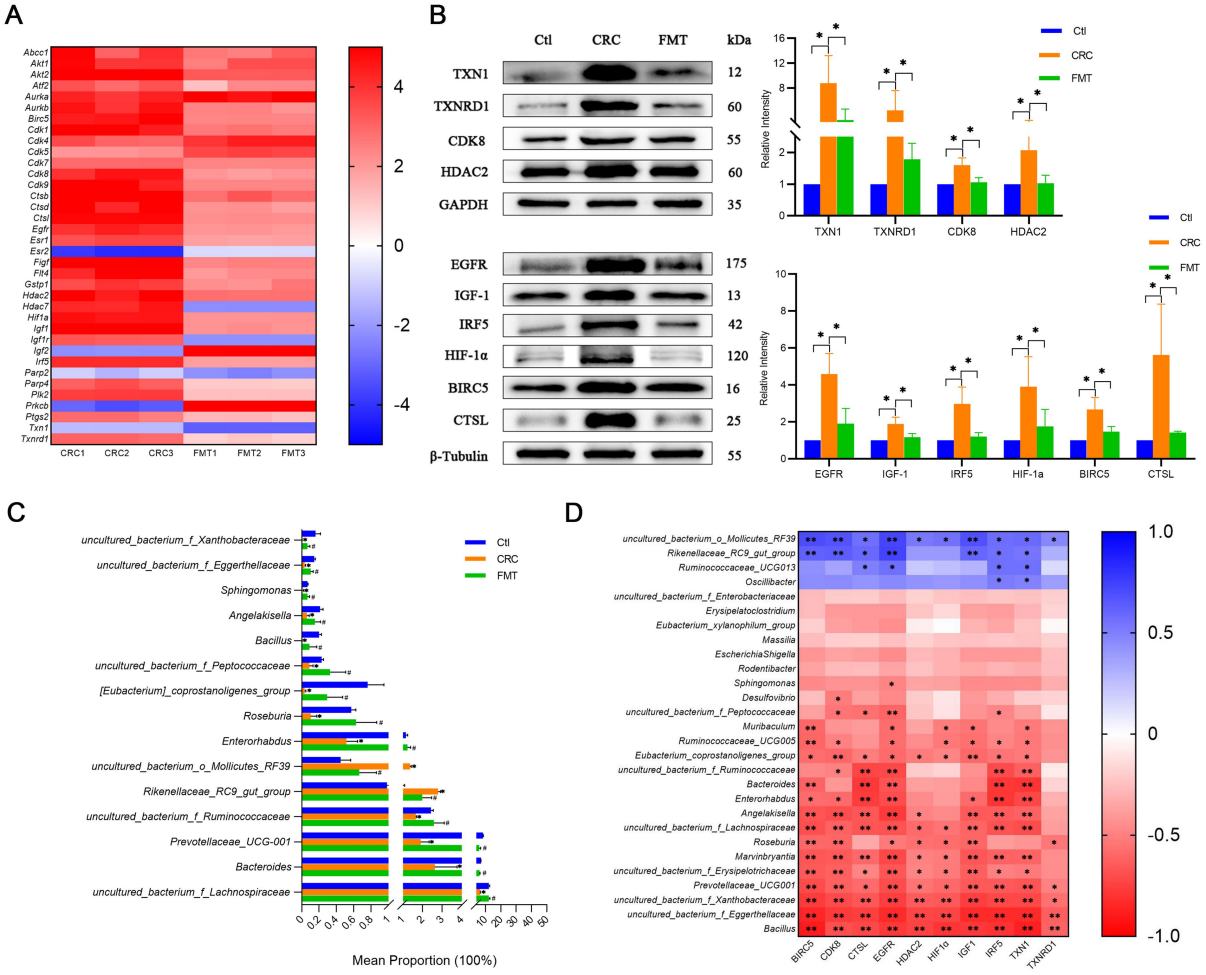
FMT inhibits the expression levels of cancer-promoting molecules. **(A)** Differentially expressed genes associated with CRC development. **(B)** Protein levels of the cancer-promoting molecules detected by western blot. ^*^p < 0.05, *n* = 3. **(C)** The abundance of intestinal microbes related to the cancer-promoting factors. ^*^p < 0.05, Ctl vs. CRC; ^#^*p* < 0.05, CRC vs. FMT, *n* = 3. **(D)** Correlation analysis between the intestinal bacteria and the molecules screened out. ^*^p < 0.05 and ^**^p < 0.01, *n* = 3.

First, to find possible targets for FMT to halt the progression of CRC, a genetic screening was done first. Results showed that 17 genes significantly differed in expression between the FMT-treated and non-treated groups, including genes involved in oxidative stress *Ptgs2*, *Txn1*, *Txnrd1*, drug resistance *Abcc1*, transcription factors *Atf2*, *Hif1a*, *Irf5*, apoptosis gene *Birc5*, gene in the cell cycle *Cdk8*, cathepsins *Ctsl*, growth factors and receptors *Egfr*, *Figf*, *Flt4*, *Igf1*, histone deacetylases *Hdac2*, poly ADP-ribose polymerases *Parp4*, protein kinases *Plk2* (Figure 3A).

At the protein level, the expression of these genes was further investigated. The thioredoxin (TXN)/thioredoxin reductase (TXNRD) system is one of the major redox control mechanisms (Holmgren and Lu, 2010). Cancer cells that express high levels of TXN1 and TXNRD1 are better able to withstand the effects of oxidative stress, proliferate, and even invade and metastasize (Zhang et al., 2017). In comparison to normal controls mice, there was a notable increase in TXN1 and TXNRD1 expression in CRC mice. Their protein expression was substantially lower in mice administered with FMT, indicating that alterations in the gut microbiota affected their expression as well as their carcinogenic characteristics (Figure 3B).

The activation of the epidermal growth factor receptor (EGFR) signaling pathway promotes the secretion of insulin-like growth factor-1 (IGF-1), which in turn induces tumor-associated macrophage polarization. Tumor-associated macrophages have been shown to accelerate the development of CRC (Zhang W. et al., 2016). An inflammatory macrophage phenotype has been demonstrated to be promoted by interferon regulatory factor 5 (IRF5), a potential intrinsic regulator of the intestinal macrophage hallmark (Corbin et al., 2020). Moreover, the IRF5-induced inflammatory response may enhance the production of hypoxia-inducible factor 1-α (HIF-1α) in dendritic cells (Hammami et al., 2015). HIF-1α is a pivotal modulator in metabolic reprogramming, which occurs in hypoxic cancer cells (Infantino et al., 2021). We then validated the protein level in CRC mice and found obvious elevations of IRF5, EGFR, IGF-1, and HIF-1α in colon tissues compared with normal mice. FMT treatment dramatically lowered their levels of protein expression (Figure 3B).

Baculoviral inhibitor of apoptosis repeat containing 5 (BIRC5) is closely linked with high-grade cancer and differentiation (Wang et al., 2022). Cyclin-dependent kinase 8 (CDK8) has been identified as an oncogene that is associated with a reduced survival rate in colorectal cancers (Broude et al., 2015). Cathepsin L (CTSL) encodes a lysosomal cysteine proteinase that regulates cancer progression. Cancer patients with up-regulated CTSL have a worse prognosis and are more likely to have aggressive metastases (Zhang L. et al., 2022). In this study, CRC mice showed considerably higher amounts of BIRC5, CDK8, and CTSL protein than normal control mice did. FMT intervention significantly reduced their elevated protein expression. This suggests that the gut microbiota influences the expression of these proteins, which in turn affects the differentiation, metastasis, and prognosis of CRC (Figure 3B).

Histone deacetylase 2 (HDAC2) has been linked to numerous biological processes, including inflammation, cancer initiation and progression, cell signaling, cellular proliferation, and gene expression control (Jo et al., 2023). In comparison to normal controls mice, HDAC2 expression was obviously up-regulated in CRC mice, as Figure 3B illustrates, and this raised trend was dramatically suppressed by using FMT.

The abundance of gut bacteria linked to these cancer-promoting targets showed significant shifts (Figure 3C). The results of the correlation analysis demonstrated a positive correlation between the expression of BIRC5, CDK8, CTSL, EGFR, HDAC2, HIF-1α, IGF1, IRF5, TXN1, and TXNRD1 and uncultured_bacterium_o_Mollicutes_RF39, Rikenellaceae_RC9_gut_group, and a negative correlation with *Bacillus*, *Marvinbryantia*, *Roseburia*, *Angelakisella*, *Enterorhabdus*, *Bacteroides*, *Muribaculum* and the genera of uncultured_bacterium_f_Eggerthellaceae, uncultured_bacterium_f_Xanthobacteraceae, Prevotellaceae_UCG-001, uncultured_bacterium_f_Erysipelotrichaceae, uncultured_bacterium_f_Lachnospiraceae, uncultured_bacterium_f_Ruminococcaceae, Eubacterium_coprostanoligenes_group, Ruminococcaceae_UCG-005, and uncultured_bacterium_f_Peptococcaceae (Figure 3D).

### 3.4 Localization of oncogenic targets in intestinal tissues

Structurally, the intestinal tissue is mainly composed of the mucosa, submucosa, muscularis propria, and serosal layer. Perhaps as a result of the mucosa’s direct exposure to the intricate interior environment of the intestine, the majority of CRC cases arise in this area. This cancer-prone site is further subdivided into the mucosal epithelium, lamina propria, and muscularis mucosa.

**Figure 4:**
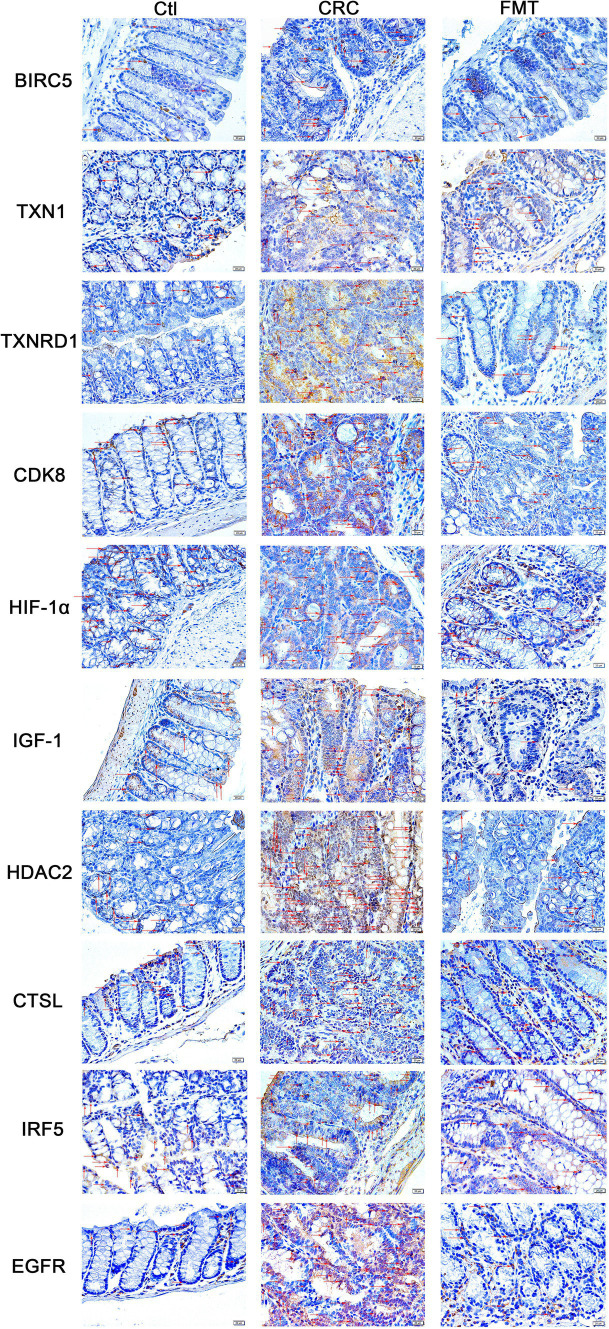
Locations of the oncogenic targets in the intestinal tissue. BIRC5, TXN1, TXNRD1, CDK8, HIF-1α, IGF-1, HDAC2, CTSL, IRF5, and EGFR were distributed in all sub-structures of the mucosa, including the mucosal epithelium, amina propria, and muscularis mucosae.

The mucosal epithelium consists of epithelial cells that secrete mucus to maintain the mucosal surface moist and shield the intestinal tissue from injury, irritation, and infection of the intestinal environment while also absorbing nutrients and water. Numerous blood and lymphatic capillaries that supply nutrients, oxygen, and aid in the removal of metabolic waste are abundant in the lamina propria. Mucosal immune protection is also supported by the lymphoid tissue and immune cells that are dispersed here. Muscularis mucosae is composed of smooth muscles, the contraction and relaxation of which contribute to the digestion and propulsion of food and regulate the volume and pressure of the mucosa. In this study, immunohistochemical staining revealed that BIRC5, TXN1, TXNRD1, CDK8, HIF-1α, IGF-1, HDAC2, CTSL, IRF5, and EGFR were not only strongly expressed within the mucosal epithelium and lamina propria but also present in a small proportion of cells within the muscularis mucosae (Figure 4). In comparison to normal control mice, the intestinal tissue of CRC animals showed disruptions in immune defense, barrier, and nutritional and metabolic pathways due to the overexpression of these molecules in the mucosa. After restoring the balance of the gut microbiota by FMT, the overexpression of these targets was significantly inhibited. Through regulating the expression of these oncogenic chemicals, the gut microbiota plays an important role in the regulation of intestinal tissue function. Sustaining the equilibrium of the gut microbiota contributes to the preservation of the typical functioning of intestinal tissue.

## 4 Discussion

In a previous work, we discovered that by disrupting the intestinal immune microenvironment, the gut microbiota significantly contributes to the regulation of CRC progression. The disruption of the intestinal microbiota in CRC mice was corrected by FMT intervention, and immune cells and inflammatory cytokines were also regulated in the intestinal tissue (Yu et al., 2023). Nevertheless, this investigation revealed that changes in the diversity and abundance of gut bacteria also impacted the expression of oncogenic molecules at both gene and protein levels, such as TXN1, TXNRD1, IRF5, EGFR, IGF-1, HIF-1α, BIRC5, CDK8, CTSL, and HDAC2. This suggests that the intestinal microbiota influences CRC development in numerous aspects and holds significant promise for its therapeutic management.

In this study, the fecal microbiota transplanted from healthy mice effectively inhibited microbial dysbiosis in CRC mice. The FMT therapy of CRC mice resulted in a considerable increase in the richness and variety of the microbial population. Principal coordinate analysis and phylogenetic analysis of the microbial sequencing data revealed that FMT intervention caused the CRC mice’s gut microbiota structure to resemble that of the normal control group.

The short-chain fatty acid butyrate, which is mostly produced by *Eubacterium*, *Roseburia*, *Lachnospiraceae*, and *Ruminococcaceae*, inhibits the growth and proliferation of cancer cells by decreasing the activity of HDACs in immune cells and colon cells and causing the expression of CDK inhibitors, which leads to cell cycle arrest in the G1 phase (Gu et al., 2023; Li and Seto, 2016; Louis et al., 2014). According to this study, FMT dramatically raised the low abundance of *Eubacterium* and *Ruminococcaceae* in CRC mice, which may encourage butyrate synthesis and in turn inhibit the activation and expression of HDAC2 and CDK8. Propionate, produced by bacteria from *Roseburia*, *Bacteroides*, and *Lachnospiraceae*, inhibits cancer progression by inducing apoptosis and cell cycle arrest through down-regulating BIRC5 expression (Louis et al., 2014; Kim et al., 2019; Bilotta and Cong, 2019). The study’s findings demonstrated that FMT restored the bacteria’s abundance in CRC mice to levels comparable to those in control mice, perhaps assisting in the downregulation of BIRC5 expression in cancer treatment-related scenarios.

Hypoxic conditions are a common characteristic of the cancer microenvironment. In these circumstances, the metabolism of cancer cells is reprogrammed, thus promoting the survival and proliferation of cancer cells and ensuring cancer progression (Infantino et al., 2021). HIF-1a is a member of the hypoxia-inducible factors family that is triggered by the gut bacteria *Yersinia enterocolitica*. It inhibits mitochondrial oxidative metabolism by lowering oxygen consumption, ensuring oxygen homeostasis in cancer tissues under hypoxia (Gardlik, 2014). TXNRD, a component of the antioxidant defense system involved in regulating the redox balance and sensing the oxidative stress state, is activated by LPS and regulated by Keap1-Nrf2/ho-1, the main regulator of various antioxidant enzymes (Li et al., 2021; Yu et al., 2021). Its primary substrate, TXN1, was likewise diminished in cells devoid of TXNRD1 (Arnér, 2021). Therefore, lowering the number of bacteria that produce LPS with FMT may aid in blocking the actions of TXNRD1 and TXN1, encourage oxidative stress-induced damage to cancer cells, and thereby stop the onset and progression of colorectal cancer.

Chronic inflammation is a major risk factor for CRC (Lucas et al., 2017). Numerous pro-inflammatory cytokines have been reported to be positively associated with the incidence of colorectal adenomas (Kim et al., 2008). Inflammatory cells have the ability to activate a significant amount of lysosomal protease CTSL, which may facilitate invasion and metastasis and result in a poor prognosis (Gomes et al., 2020). The expression of HDAC2 in macrophages induced by LPS has a positive effect on inducing proinflammatory cytokine expression (Wu et al., 2018). Inflammation in the intestinal tract increases EGFR expression (Hardbower et al., 2017). The activation of the EGFR signaling pathway enhances IGF-1 secretion and directly drives M2 macrophage polarization, resulting in an immunosuppressor effect and promoting the development of cancer (Yang et al., 2021). One of the key players in inflammation among the IRF family members is IRF5. IRF5 is temporarily expressed by inflammatory stimuli in macrophages, and IRF5 overexpression causes proinflammatory cytokines like IL-6, IL-12, IL-23, and TNF-α to be produced (Krausgruber et al., 2011). This increases inflammation, accelerates the development of CRC, and interferes with regular bowel movements. On the other hand, guanosine produced by *Roseburia* and *Enterorhabdus* has an anti-inflammatory impact by lowering the expression of TNF-α, IL-1β, and IL-6 (Zhang H. et al., 2022; Zizzo et al., 2018). Additionally, uncultured bacteria f_Peptococcaceae metabolize tryptophan to produce indole acrylic acid, a sign of an anti-inflammatory immune response (Wlodarska et al., 2017). *Bacillus subtilis* administration alleviates systemic inflammation in mice by balancing pro- and anti-inflammatory factors in the gut (Zhang H.-L. et al., 2016). Changes to these oncogenic molecules are associated with the process by which intestinal inflammation expedites the progression of CRC, as demonstrated by the correction of these intestinal bacterial abnormalities by FMT in our study.

Based on the previously mentioned findings and examination, applying FMT to reverse intestinal dysbiosis in CRC mice and maintain intestinal environment homeostasis resulted in a reduction in the expression of oncogenic molecules. These noteworthy anti-cancer effects offered a theoretical foundation for the treatment and prevention of CRC. The strong relationships between the expression of some carcinogenic chemicals and the quantity of various bacteria were also examined in this work; further research is needed to determine the mechanisms underlying FMT’s inhibition of CRC progression.

## Data Availability

The data on intestinal microbiota 16S rDNA are available at the National Center for Biotechnology Information (NCBI) (https://www.ncbi.nlm.nih.gov/) with the following accession number: PRJNA 949363. The raw data supporting the conclusion of this article will be made available by the authors without undue reservation.
