# Supplementary figures and images for: Suppressed oncogenic molecules involved in the treatment of colorectal cancer by fecal microbiota transplantation
Source: Front Microbiol. 2024 Nov 13;15:1451303. doi: 10.3389/fmicb.2024.1451303 (PMC11605715; doi:10.3389/fmicb.2024.1451303)

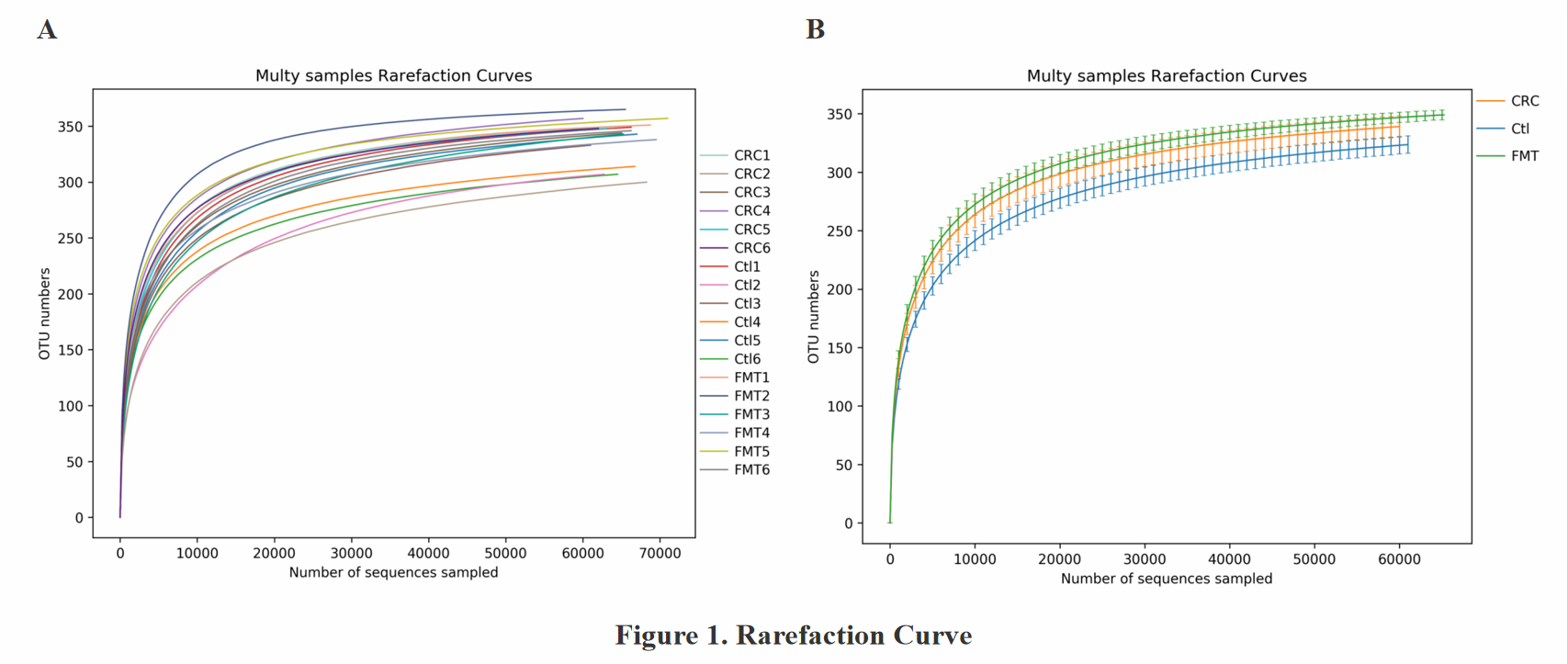

Supplement: Supplementary file 1 [file Image_1.png]
