# Supplementary material for: Suppressed oncogenic molecules involved in the treatment of colorectal cancer by fecal microbiota transplantation
Source: Front Microbiol. 2024 Nov 13;15:1451303. doi: 10.3389/fmicb.2024.1451303 (PMC11605715; doi:10.3389/fmicb.2024.1451303)
Supplement: Supplementary file 2 [file Table_1.docx]

**Table 1. sequencing data of intestinal microbiota**

| **Sample ID** | **Raw Reads** | **Clean Reads** | **Effective Reads** | **AvgLen(bp)** |
| --- | --- | --- | --- | --- |
| Ctl1 | 79,742 | 79,303 | 77,687 | 421 |
| Ctl2 | 80,339 | 79,899 | 78,277 | 422 |
| Ctl3 | 80,393 | 80,014 | 78,146 | 421 |
| Ctl4 | 79,684 | 79,280 | 77,668 | 420 |
| Ctl5 | 80,131 | 79,720 | 78,411 | 421 |
| Ctl6 | 79,979 | 79,557 | 77,904 | 419 |
| CRC1 | 80,120 | 79,665 | 78,358 | 419 |
| CRC2 | 79,698 | 79,239 | 77,228 | 422 |
| CRC3 | 79,813 | 79,341 | 77,397 | 418 |
| CRC4 | 80,105 | 79,664 | 77,622 | 418 |
| CRC5 | 80,159 | 79,711 | 78,074 | 420 |
| CRC6 | 79,812 | 79,399 | 77,607 | 419 |
| FMT1 | 79,873 | 79,446 | 77,857 | 415 |
| FMT2 | 80,504 | 80,085 | 78,461 | 415 |
| FMT3 | 79,366 | 78,992 | 77,909 | 421 |
| FMT4 | 80,147 | 79,702 | 78,412 | 414 |
| FMT5 | 80,184 | 79,767 | 78,539 | 417 |
| FMT6 | 80,356 | 79,934 | 78,332 | 420 |
